# Association between parents’ country of birth and multicultural adolescents’ psychological well-being in South Korea: A study on depression, worries, life satisfaction, and social withdrawal

**DOI:** 10.1371/journal.pmen.0000356

**Published:** 2025-06-18

**Authors:** Abdullatif Ghafary, Jaeyong Shin, Sang Sook Beck, Jieun Jang, Rajaguru Vasuki, So Yoon Kim

**Affiliations:** 1 Department of Medical Law and Ethics, Yonsei University, Seoul, South Korea; 2 Department of Preventive Medicine, Yonsei University, Seoul, South Korea; 3 Graduate School of Public Health, Yonsei University, Seoul, South Korea; 4 Department of Preventive Medicine, Dongguk University, Seoul, South Korea; 5 Department of Healthcare Management, Yonsei University, Seoul, South Korea; Universiti Brunei Darussalam Pengiran Anak Puteri Rashidah Sa'adatul Bolkiah Institute of Health Sciences, BRUNEI DARUSSALAM

## Abstract

The purpose of this study was to examine the association between parents’ country of birth and psychological well-being of multicultural adolescents in Korea, a country with a predominantly homogenous population. This study used data from the 8th wave of the Multicultural Adolescents Panel Study (MAPS) conducted by the National Youth Policy Institute (NYPI). The participants included 1,147 multicultural adolescents (561 males, 586 females, mean age = 16.96 years). Adolescents whose mothers were born abroad, particularly those whose mothers were Chinese or Filipino, exhibited higher odds of experiencing depression (OR=1.13; 95% CI, 0.50–2.56) compared to those with native Korean mothers. Compared with male adolescents, female respondents were more likely to experience depression (OR = 1.28; 95% CI, 0.99–1.66), worries (OR = 1.98; 95% CI, 1.51–2.59), and lower life satisfaction (OR = 0.75; 95% CI, 0.56–1.01). There was an association between mothers’ level of education and adolescents’ depression, with higher education levels corresponding to lower depression rates (0R = 0.31; 95% CI, 0.13–0.72). These findings have important implications for addressing unique psychological needs in the context of multicultural adolescents, integrated with parental and socioeconomic factors. More support and policy measures should be taken to increase psychological well-being in this growing demographic segment.

## Introduction

Within the past two decades, the population of multicultural families, which are composed of married immigrants or foreigners with Korean citizenship, has significantly increased [1]. For clarity, throughout this manuscript, ‘Korea’ refers specifically to South Korea. The number of students from multicultural families also rose by 7.4% in 2020 to approximately 147,000 students, while the overall number of students in Korea decreased. Adolescents from multicultural families currently constitute 2.2% of the total adolescent population in Korea [1]. Korean adolescents are experiencing significant mental health issues, including depression and suicidal behavior. These issues are influenced by gender, age, and family background [2]. Moreover, multicultural adolescents face additional stressors, such as acculturative stress and discrimination, which escalate these issues [3,4]. The social environment, mental health interventions, and exposure to trauma events play vital roles in life satisfaction [5]. Similarly, for multicultural adolescents, their relationships with parents and the support they receive from the community are crucial for their psychological well-being [6]. Parental background is associated with adolescents’ psychological well-being, particularly in multicultural context. Research indicated that sociocultural factors, including parental nationality, education, and socioeconomic status, affect adolescent mental health outcomes [7]. A study on multicultural adolescents in Korea found that acculturative stress, self-esteem, family support, and economic status were the key predictors of mental health challenges [7]. These findings align with study where adolescents from immigrant backgrounds often experience higher rates of anxiety and depression as a consequence of cultural adaptation struggles [8]. The COVID-19 pandemic has intensified mental health problems, leading to increased depression and anxiety. The stress of fitting into a new culture has contributed to some adolescents withdrawing socially [4,9]. Acculturation theory suggests that multicultural adolescents must navigate dual cultural identities, and this can lead to stress and social withdrawal [1]. Studies indicated that language barriers, discrimination, and parental expectations contribute to elevated psychological distress in multicultural adolescents [8]. Additionally, research on gender differences in depression among multicultural adolescents in Korea found that female adolescents reported higher depression rates compared to male adolescents, emphasizing the need for gender-based interventions [8]. The quality of the parent-child relationship is directly associated with adolescents’ mental health outcomes, such as depression and worries [10]. Geographical and environmental elements, along with the family’s socioeconomic status, are linked with adolescents’ risky behaviors [10]. Studies have shown that, compared with their urban counterparts, adolescents in rural areas are more likely to engage in risky behaviors, such as substance use (i.e., drugs, alcohol, and tobacco), bringing weapons to school, and having sexual intercourse [11]. These risky behaviors increase family stress and impact parenting, given that in rural areas, access to mental health care services is limited [12]. The increase in multicultural families has brought about considerable changes in adolescence and has led to an increase in psychological problems among multicultural families. Adolescents undergo physical, emotional, and social changes, leading to impulsive thoughts and unstable psychological issues such as conflict, tension, and stress among middle and late adolescents, especially among multicultural families [8]. Adolescence is a dynamic period in human development. In addition to accompanying physiological changes, it is characterized by significant psychosocial growth [13]. This period is critical for adolescents, as they begin to explore their identities and positions in society while they grow up physically, psychologically, and socially [14]. The challenges faced by multicultural adolescents in school, the effects of the host country culture, and their relationships with parents can directly and indirectly affect their psychological well-being [15]. Studies indicate that children of immigrants tend to exhibit a higher risk of mental health problems, such as depression and suicidal behavior [16]. In 2020, Korea had the highest suicide rate among OECD member countries [17], revealing the need to act regarding the psychological status of adolescents.

Among the critical factors influencing multicultural adolescents in Korea, the presence of acculturation stress could be associated with increased depressive symptoms and decreased self-esteem [18]. Parental factors, country of birth, level of education, and occupation are some of the significant determining factors among adolescents [15,19].

Addressing the psychological well-being of multicultural adolescents is crucial for promoting social sustainability, and the psychological well-being of future generations is rooted in ethnic diversity [20]. Since adolescence is an irregular period in which biological, cognitive, and social changes occur rapidly, adolescents are vulnerable to developing depression and suicidal symptoms [14]. Previous studies have shown that depression in adolescence is likely to transpire into depression during adulthood [21]. Given these challenges, it is important to understand how parental factors specifically, parent’s country of birth is associated with the psychological well-being of multicultural adolescents. Multicultural adolescents in Korea include children from international marriages, immigrant adolescents, and foreign children. This study focuses mainly on children from families with international marriages. To examine these associations, the study assesses depression, worries, life satisfaction, and social withdrawal, while also considering parental education, occupation and socioeconomic status.

## Methodology

### Ethics statement

The study data were obtained from the National Youth Policy Institute (NYPI) after submitting the research plan, and all personal identifiers were excluded to ensure confidentiality.

Although formal IRB approval was not required by NYPI for this specific analysis, informed consent was originally obtained from all participants and their guardians at the time of the initial data collection. All procedures adhered to ethical guidelines, including confidentiality and data protection, as outlined in the Declaration of Helsinki.

### Data set and sample

**Fig 1:**
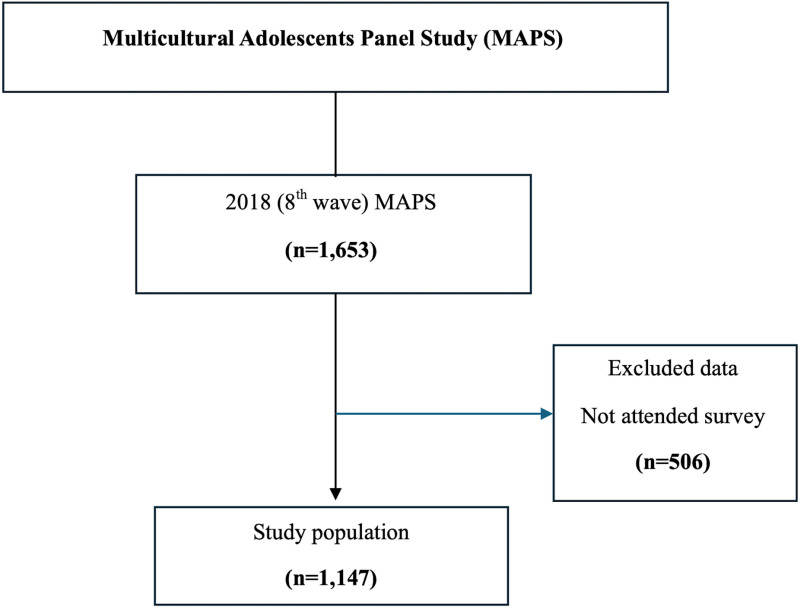
Flow chart.

For this cross-sectional study, we used raw data from the 8^th^ wave of the Multicultural Adolescents Panel Study (MAPS), conducted by the National Youth Policy Institute (NYPI) in Korea, the dataset consists of 1,147 multicultural adolescents (see Fig 1). MAPS was initiated to track the developmental processes of multicultural adolescent [22]. Since the dataset was pre-collected through annual surveys, no independent sampling was performed. Instead, we extracted and structured the dataset to align with our research objectives. For additional details, see S1 Data: Full dataset and codebook. Additionally, for better understanding of the dataset, see S1 Text: Readme. Sample selection was based on data indicating that 95% of the multicultural adolescents in Korea has a foreign mother and a Korean father. Since 2011, annual surveys have investigated 4^th^ grade elementary school students (aged 9–10 years) and their mothers. Further details on the MAPS dataset are available at https://www.nypi.re.kr/archive.

### Data analysis

Data were analyzed using SPSS software version 26.0. Descriptive statistics (percentages, frequencies) categorize the demographics of multicultural adolescents based on gender, age, parental nationality, education, and socioeconomic status. Normality and homogeneity tests were conducted using kurtosis, skewness, and Levene’s test.

**Fig 2:**
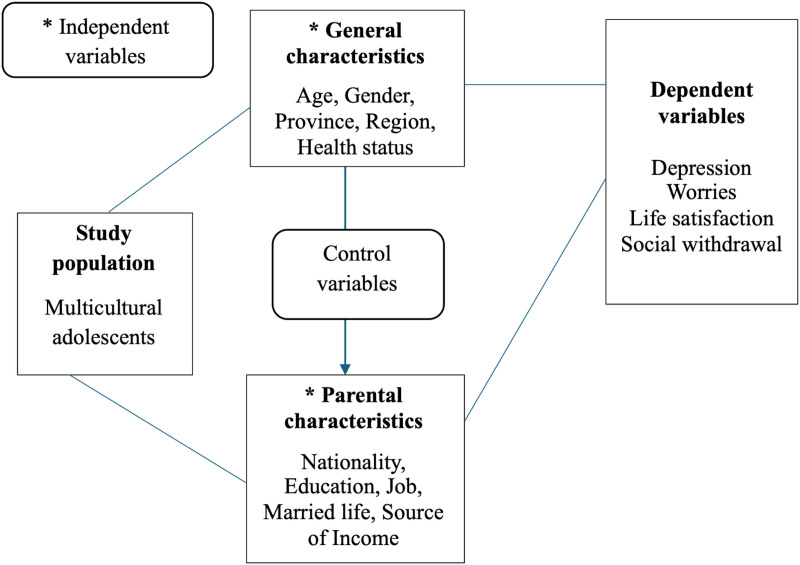
Conceptual framework.

Multiple logistic regression examined associations between depression, worries, life satisfaction, and social withdrawal in relation to parents’ country of birth, age, gender, education level, occupation, marital status, socioeconomic status, and main source of income. Fisher’s exact test was used for categorical comparisons assumptions regarding data normality, homogeneity, and validity (see Fig 2).

## Results

### General characteristics

Among the 1,147 participants, 1,109 (96.7%) were foreign mothers, and 1,107 (96.5%) were Korean fathers. The number of participants from the Philippines (25.1%) and China (24.4%) was the highest among foreign mothers. In the current study, 70% of the participants lived in cities, whereas 30% lived in rural areas. A majority (75.2%) answered that fathers were the primary source of income, and 51.5% stated that their families encountered challenging socioeconomic conditions. In addition, 49.7% of the respondents experienced social withdrawal, and 53.7% experienced depression. A total of 36.0% of the respondents were dissatisfied with their future, socioeconomic status, studies, school, work, and physical and psychological well-being. Most parents were high school graduates (see Table 1).

**Table 1:** Characteristics of study participants (N = 1,147).

Variables	Categories	N	%
**Mother’s country of birth**	Korean	38	3.3
	Non-Korean	1109	96.7
**Mother’s nationality**	Korean	38	3.3
	Chinese	280	24.4
	Vietnamese	25	2.2
	Filipino	288	25.1
	Other	516	45.0
**Father’s country of birth**	Korean	1107	96.5
	Non-Korean	40	3.5
**Communication with parents**	Korean	776	67.7
	Other language	371	32.3
**Gender**	Male	561	48.9
	Female	586	51.1
**province**	Capital	397	34.6
	Others	750	65.4
**Region**	Urban	803	70.0
	Rural	344	30.0
**Age**	16	84	7.3
	17	1023	89.2
	18	36	3.1
	19	3	0.3
	20	1	0.1
**Mother’s education**	Middle school or less	45	3.9
	High school	541	47.2
	Undergraduate & over	474	41.3
**Father’s education**	Middle school or less	360	31.4
	High school	594	51.8
	Undergraduate & over	193	16.8
**Mother’s job**	Professional or experts	358	31.2
	Technician or labor	201	17.5
	Others	588	51.3
**Father’s job**	Professional or experts	207	18.0
	Technician or labor	410	35.7
	Others	530	46.2
**Parents` married life**	Married	1059	88.5
	Others Ω	138	11.5
**The main source of income**	Father	862	75.2
	Mother	217	18.9
	Others	68	5.9
**Socioeconomic status**	Difficult	616	51.5
	Good	559	46.7
	Missing	22	1.8
**Health status**	Healthy	1038	90.5
	Unhealthy	109	9.5
**Self-esteem**	Not good	1025	89.4
	Good	122	10.6
**Life satisfaction**	Not good	287	25.0
	Good	860	75.0
**Depression**	Not depressed	531	46.3
	Depressed	616	53.7
**Social withdrawal**	No	577	50.3
	Yes	570	49.7
**Worries and concerns**	No	734	64.0
	Yes	413	36.0
**Academic achievement**	Below average	399	34.8
	Above average	748	65.2

### Associations of depression and worries with the general characteristics of multicultural adolescents

Adolescents whose mothers were Filipino (45.83% depression, 36.8% concern) or Chinese (51.07% depression, 36.1% concern) showed greater depression and concern than those whose mothers were Korean. Higher rates of depression (53.21%) and concern (35.7%) were also found among adolescents whose fathers were Korean. Compared with male participants, female participants presented higher rates of depression (57.51%) and concern (44.0%) (see Table 2).

**Table 2:** Association of depression and worries with general characteristics of multicultural adolescents (N = 1,147).

Variables	Categories	Depression					Worries				
		Yes		No		p*	Yes		No		p*
		N	%	N	%		N	%	N	%	
Mother’s nationality	Korean	25	65.79	13	34.21	0.003	8	32.0	17	68.0	0.972
	Chinese	143	51.07	137	48.93		101	36.1	179	63.9	
	Vietnamese	12	48.00	13	52.00		15	39.5	23	60.5	
	Filipino	132	45.83	156	54.17		106	36.8	182	63.2	
	Other	304	58.91	212	41.09		183	35.5	333	64.5	
Father’s country of birth	Korean	589	53.21	518	46.79	0.051	395	35.7	712	64.3	0.15
	Non-Korean	27	67.50	13	32.50		18	45.0	22	55.0	
Gender	Male	279	49.73	282	50.27	0.005	155	27.6	406	72.4	<.001
	Female	337	57.51	249	42.49		258	44.0	328	56.0	
Region	Urban	449	55.92	354	44.08	0.013	92	11.5	711	88.5	0.100
	Rural	167	48.55	177	51.45		30	8.7	314	91.3	
Age	16	46	54.76	38	45.24	0.249	32	38.1	52	61.9	0.052
	17	544	53.18	479	46.82		360	35.2	663	64.8	
	18+	26	69.44	14	30.56		21	50.0	19	50.0	
Province	Capital	269	67.76	128	32.24	<.001	153	38.5	244	61.5	0.109
	Other	347	46.27	403	53.73		260	34.7	490	65.3	
Mother’s education	Middle school or less	37	82.22	8	17.78	<.001	22	48.9	23	51.1	0.102
	High school	281	51.94	260	48.06		195	36.0	346	64.0	
	Undergraduate & over	259	54.64	215	45.36		158	33.3	316	66.7	
Father’s education	Middle school or less	175	48.61	185	51.39	0.058	124	34.4	236	65.6	0.606
	High school	330	55.56	264	44.44		222	37.4	372	62.6	
	Undergraduate & over	111	57.51	82	42.49		67	34.7	126	65.3	
Mather’s job	Professional or experts	205	57.26	153	42.74	0.106	121	33.8	237	66.2	0.573
	Technician or labor	113	56.22	88	43.78		75	37.3	126	62.7	
	Others	298	50.68	290	49.32		217	36.9	371	63.1	
Father’s job	Professional or experts	110	53.14	97	46.86	0.838	70	33.8	137	66.2	0.143
	Technician or labor	224	54.77	185	45.23		163	39.8	247	60.2	
	Others	281	53.02	249	46.98		180	34.0	350	66.0	
The main source of income	Father	446	51.74	416	48.26	0.043	320	37.1	542	62.9	0.369
	Mother	126	58.06	91	41.94		72	33.2	145	66.8	
	Others	44	64.71	24	35.29		21	30.9	47	69.1	
Communication with parents	Korean	401	51.68	375	48.32	0.027	256	33.0	520	67.0	<.001
	Other	215	57.95	156	42.05		157	42.3	214	57.7	
Socioeconomic status	Difficult	340	56.57	261	43.43	0.024	224	37.3	377	62.7	0.191
	Good	276	50.55	270	49.45		189	34.6	357	65.4	
Health status	Healthy	538	51.83	500	48.17	<.001	346	33.3	692	66.7	<.001
	Unhealthy	78	71.56	31	28.44		67	61.5	42	38.5	
Academic achievement	Below average	251	62.91	148	37.09	<.001	166	41.6	233	58.4	0.002
	Above average	365	48.80	383	51.20		247	33.0	501	67.0	

Urban adolescents had a higher rate of depression (55.92%) than did rural adolescents (48.55%), although only 11.5% of urban adolescents worried about their future. Adolescents whose parents had secondary education were more depressed and worried. Higher rates of depression and worries were associated with adolescents whose parents were unemployed, students, or housewives.

### Association of life satisfaction and social withdrawal with the general characteristics of multicultural adolescents

Life satisfaction rates were comparable across different mothers’ nationalities. Those with Chinese mothers (77.1% life satisfaction, 52.9% social withdrawal) and Filipino mothers (74.3% life satisfaction, 47.6% social withdrawal) showed greater social withdrawal than did those with Korean mothers (78.9% life satisfaction, 68.4% social withdrawal). However, these differences were not statistically significant (p = 0.834 and p = 0.088, respectively). Compared with non-Korean fathers, adolescents with Korean fathers scored higher in terms of life satisfaction (74.9%) but were more socially withdrawn (49.6%), with a significant difference in social withdrawal (p = 0.008) (see Table 3).

**Table 3:** Association of life satisfaction and social withdrawal with general characteristics of multicultural adolescents (N = 1,147).

Variables	Categories	Life Satisfaction					Social Withdrawal				
**Yes**		**No**		p*	**Yes**		**No**		p*
	**N**	**%**	**N**		**%**	**N**	**%**	**N**	**%**	
**Mother’s nationality**	Korean	30	78.9	8	21.1		26	68.4	12	31.6	0.088
	Chinese	216	77.1	64	22.9		148	52.9	132	47.1	
	Vietnamese	19	76.0	6	24.0	0.834	15	60.0	10	40.0	
	Filipino	214	74.3	74	25.7		137	47.6	151	52.4	
	other	381	73.8	135	26.2		251	48.6	265	51.4	
**Father’s country of birth**	Korean	829	74.9	278	25.1	0.436	549	49.6	558	50.4	0.008
	Non-Korean	31	77.5	9	22.5		28	70.0	12	30.0	
**Gender**	Boys	439	78.3	122	21.7	0.007	292	52.0	269	48.0	0.136
	Girls	421	71.8	165	28.2		285	48.6	301	51.4	
**Region**	Urban	582	72.5	221	27.5	0.002	402	50.1	401	49.9	0.426
	Rural	278	80.8	66	19.2		175	50.9	169	49.1	
**Age**	16	60	71.4	24	28.6	0.46	41	48.8	43	51.2	0.402
	17	774	75.7	249	24.3		514	50.2	509	49.8	
	18 +	26	65.0	14	35.0		22	55.0	18	45.0	
**Province**	Capital	262	66.0	135	34.0	<.001	197	49.6	200	50.4	0.392
	Other	598	79.7	152	20.3		380	50.7	370	49.3	
**Mother’s education**	Middle school or less	29	64.4	16	35.6		17	37.8	28	62.2	0.02
	High school	415	76.7	126	23.3	0.105	258	47.7	283	52.3	
	Undergraduate & over	345	72.8	129	27.2		259	54.6	215	45.4	
**Father’s education**	Middle school or less	286	79.4	74	20.6		169	46.9	191	53.1	<.001
	High school	423	71.2	171	28.8	0.009	292	49.2	302	50.8	
	Undergraduate & over	151	78.2	42	21.8		116	60.1	77	39.9	
**Mather’s job**	Professional or experts	276	77.1	82	22.9		185	51.7	173	48.3	0.17
	Technician or labor	147	73.1	54	26.9	0.508	89	44.3	112	55.7	
	Others	437	74.3	151	25.7		303	54.0	258	46.0	
**Father’s job**	Professional or experts	167	80.7	40	19.3		111	53.6	96	46.4	0.125
	Technician or labor	317	77.3	93	22.7	0.009	190	46.3	220	53.7	
	Others	376	70.9	154	29.1		276	52.1	254	47.9	
**The main source of income**	Father	646	74.9	216	25.1	0.803	444	51.5	418	48.5	0.359
	Mother	165	76.0	52	24.0		102	47.0	115	53.0	
	Others	49	72.1	19	27.9		31	45.6	37	54.4	
**Communication with parents**	Korean	592	76.3	184	23.7	0.080	399	51.4	377	48.6	0.152
	Other	268	72.2	103	27.8		178	48.0	193	52.0	
**Socioeconomic status**	Difficult	447	74.4	154	25.6	0.335	308	51.2	293	48.8	0.271
	Good	413	75.6	133	24.4		269	49.3	277	50.7	
**Health status**	Healthy	791	76.2	247	23.8	0.003	542	52.2	496	47.8	<.001
	Unhealthy	69	63.3	40	36.7		35	32.1	74	67.9	
**Academic achievement**	Below average	256	64.2	143	35.8	<.001	152	38.1	247	61.9	<.001
	Above average	604	80.7	144	19.3		425	56.8	323	43.2	

A comparison by gender revealed that boys experienced greater life satisfaction (78.3%, p = 0.007), with no difference in social withdrawal (52.0% boys, 48.6% girls, p = 0.136). Compared with rural adolescents, urban adolescents reported lower life satisfaction (72.5%, p = 0.002) (80.8%), but there was no difference in social withdrawal (50.1% urban, 50.9% rural, p = 0.426).

Parents’ education influences both life satisfaction and social withdrawal. Adolescents whose mothers had middle school education or below experienced lower life satisfaction (64.4%, p = 0.02) and greater social withdrawal (37.8%) than did those whose mothers had high school education (76.7%, 47.7%) or undergraduate education or above (72.8%, 54.6%).

Adolescents whose fathers had a high school education reported the lowest levels of life satisfaction (72.2%, p = 0.009) and experienced greater social withdrawal (49.2%). This was in contrast with those whose fathers had middle school or less education (79.4% life satisfaction, 46.9% social withdrawal) and those whose fathers had undergraduate or higher education (78.2% life satisfaction, 60.1% social withdrawal, p < 0.001). Being in good physical and psychological well-being was a good factor for higher life satisfaction (76.2%, p = 0.003) and lower social withdrawal (52.2%, p < 0.001) than being in poor health (63.3% life satisfaction, 32.1% social withdrawal).

Academic performance also played a role, with above-average achievers showing greater life satisfaction (80.7%, p < 0.001) and lower social withdrawal (56.8%) than below-average performers did (64.2% life satisfaction, 38.1% social withdrawal).

### Multiple logistic regression analysis of factors associated with multicultural adolescents’ psychological wellbeing

Adolescents with non-Korean fathers had greater odds of worries (OR=11.42; 95% CI, 1.24–105.35; p = 0.032). Female adolescents exhibited higher odds of depression (OR=1.98; 95% CI, 0.99–1.66; p = 0.040) and worries (OR=1.98; 95% CI, 1.51–2.59; p < 0.001) and lower odds of life satisfaction (OR=0.75; 95% CI, 0.56–1.01; p = 0.057) (see Table 4).

**Table 4:** Multiple logistic regression analysis of associated factors and multicultural adolescents’ psychological well-being (N = 1,147).

Variables	Categories	Depression				Worries				Life Satisfaction				Social Withdrawal			
		OR	95% (CI)		*p*	OR	95% (CI)		*p*	OR	95% (CI)		*p*	OR	95% (CI)		*p*
**Mother’s nationality**	Filipino	1.00				1.00				1.00				1.00			
	Chinese	1.13	0.50	2.56	0.769	1.20	0.50	2.88	0.684	1.07	0.41	2.78	0.896	1.34	0.58	3.08	0.494
	Korean	2.08	0.74	5.85	0.163	1.39	0.48	4.01	0.547	1.18	0.36	3.95	0.783	0.69	0.24	1.98	0.494
	Vietnamese	0.92	0.40	2.08	0.835	1.24	0.52	2.97	0.632	0.91	0.35	2.37	0.852	1.65	0.72	3.80	0.237
	Other	1.55	0.70	3.47	0.283	1.17	0.49	2.76	0.724	0.89	0.35	2.28	0.810	1.58	0.70	3.59	0.271
**Father’s country of birth**	Korean	1.00				1.00				1.00				1.00			
	Non-Korean	1.88	0.26	13.47	0.530	11.42	1.24	105.35	0.032	0.78	0.13	4.74	0.785	0.44	0.08	2.58	0.363
**Gender**	Male	1.00				1.00				1.00				1.00			
	Female	1.28	0.99	1.66	0.040	1.98	1.51	2.59	<.001	0.75	0.56	1.01	0.057	1.11	0.86	1.43	0.408
**Region**	Urban	1.00				1.00				1.00				1.00			
	Rural	0.97	0.71	1.32	0.843	1.06	0.77	1.46	0.730	1.20	0.82	1.74	0.350	0.94	0.69	1.27	0.679
**Mother’s education**	Undergraduate & over	1.00				1.00				1.00				1.00			
	Middle school or less	0.31	0.13	0.72	0.007	0.56	0.28	1.13	0.104	1.20	0.59	2.45	0.616	0.63	0.32	1.24	0.153
	High school	0.32	0.14	0.77	0.010	0.47	0.23	0.97	0.040	0.89	0.42	1.88	0.762	0.46	0.22	0.93	0.030
**Father’s education**	Undergraduate & over	1.00				1.00				1.00				1.00			
	Middle school or less	1.12	0.82	1.52	0.468	1.18	0.85	1.62	0.324	0.59	0.41	0.85	0.004	0.86	0.64	1.16	0.332
	High school	1.16	0.74	1.84	0.516	1.21	0.75	1.94	0.441	0.81	0.48	1.39	0.448	0.62	0.39	0.97	0.035
**Mother’s job**	Professional or experts	1.00				1.00				1.00				1.00			
	Technician or labor	0.80	0.59	1.08	0.148	1.20	0.88	1.65	0.243	0.74	0.53	1.05	0.093	0.91	0.68	1.22	0.527
	Others	1.14	0.75	1.72	0.550	1.02	0.67	1.57	0.923	0.62	0.39	0.99	0.053	1.23	0.82	1.85	0.308
**Father’s job**	Professional or experts	1.00				1.00				1.00				1.00			
	Technician or labor	1.11	0.76	1.64	0.588	1.01	0.68	1.51	0.955	0.56	0.36	0.88	0.011	0.76	0.52	1.11	0.162
	Others	1.40	0.92	2.14	0.114	1.37	0.89	2.10	0.154	0.75	0.46	1.23	0.248	0.96	0.64	1.44	0.840
**The main source of income**	Father	1.00				1.00				1.00				1.00			
	Mother	1.79	1.00	3.19	0.039	0.77	0.42	1.39	0.380	1.10	0.60	2.02	0.762	1.55	0.90	2.69	0.115
	Others	1.43	1.01	2.04	0.044	0.77	0.53	1.10	0.153	0.98	0.66	1.45	0.908	1.21	0.86	1.70	0.272
**Communication with parents**	Korean	1.00				1.00				1.00				1.00			
	Other	1.13	0.84	1.50	0.422	1.63	1.21	2.18	<.001	0.86	0.62	1.20	0.378	1.27	0.95	1.68	0.102
**Socioeconomic status**	Difficult	0.76	0.59	0.99	0.042	0.79	0.61	1.04	0.093	1.24	0.92	1.66	0.157	1.06	0.82	1.36	0.670
	Good	1.00				1.00				1.00				1.00			
**Health status**	Healthy	1.00				1.00				1.00				1.00			
	Unhealthy	2.12	1.32	3.40	0.002	3.40	2.19	5.27	<.001	0.54	0.35	0.86	0.008	2.07	1.33	3.23	<.001
**Academic achievement**	Below average	0.55	0.42	0.73	<.001	0.66	0.50	0.87	0.004	2.62	1.94	3.54	<.001	0.48	0.36	0.62	<.001
	Above average	1.00				1.00				1.00				1.00			

Higher mothers’ education was linked to lower odds of depression (middle school: OR=0.31; 95% CI, 0.13–0.72, p = 0.007; high school: OR=0.32; 95% CI, 0.14–0.77, p = 0.010), worries (high school: OR=0.47; 95% CI, 0.23–0.97, p = 0.040), and social withdrawal (high school: OR=0.46; 95% CI, 0.22–0.93, p = 0.030).

Fathers’ education level influenced life satisfaction (middle school: OR=0.59; 95% CI, 0.41–0.85; p = 0.004) and social withdrawal (high school: OR=0.62; 95% CI, 0.39–0.97; p = 0.035).

Children of labor workers had lower odds of life satisfaction (OR=0.56; 95% CI, 0.36–0.88; p = 0.011). Students who derived income from mothers had greater odds of depression (OR=1.79; 95% CI, 1.00–3.19; p = 0.039) and worries (OR=1.43; 95% CI, 1.01–2.04; p = 0.044).

Using non-Korean languages at home increased the odds of worries (OR=1.63; 95% CI, 1.21–2.18; p < 0.001). Unhealthy students had greater odds of depression (OR=2.12; 95% CI, 1.32–3.40; p = 0.002), worries (OR=3.40; 95% CI, 2.19–5.27; P < 0.001), social withdrawal (OR=2.07; 95% CI, 1.33–3.23; p < 0.001), and lower life satisfaction (OR=0.54; 95% CI, 0.35–0.86; p = 0.008).

Below-average academic achievement was linked to lower odds of depression (OR=0.55; 95% CI, 0.42–0.73; p < 0.001), worries (OR=0.66; 95% CI, 0.50–0.87; p = 0.004), and social withdrawal (OR=0.48; 95% CI, 0.36–0.62; p < 0.001) but higher life satisfaction (OR=2.62; 95% CI, 1.94–3.54; p < 0.001).

## Discussion

This study investigated how parental factors specifically, parent’s country of birth is associated with the psychological well-being of multicultural adolescents in Korea, with a specific focus on depression, worries, life satisfaction, and social withdrawal. The sample included 1,147 adolescents, with the majority having foreign-born mothers and Korean fathers. The findings indicated that adolescents whose mothers were Chinese or Filipino were more likely to experience greater depression, worries, and social withdrawal. While mothers’ nationalities made minimal association in life satisfaction rates, adolescents of Korean mothers reported marginally greater life satisfaction rate compared to their Filipino and Chinese counterparts. Given the small sample size of Korean mothers (N = 38), this comparison should be viewed cautiously. Similarly, having a foreign-born father was linked to increased worries. This could be explained by several factors. Adolescents of immigrants experience many conflicts and unfamiliar situations as they grow up in a dual culture where the values and attitudes of their fathers and mothers are different. Gender differences revealed that females had greater odds of depression, worries and lower life satisfaction. Higher parental education, particularly among mothers, was associated with lower odds of depression and worries. Fathers’ occupations had a noticeable association on life satisfaction, with adolescent of labor workers reporting lower life satisfaction. Additionally, adolescents with mothers from developing countries tended to have greater depressive symptoms. Many immigrants from these countries are often employed in low-paying, difficult, and undesirable jobs that Koreans refuse, which may be associated with lower socioeconomic status. Language used to communicate with parents is associated with language competency. In this study, we found that adolescents who spoke non-Korean languages at home were more likely to have worries and concerned about their lives. Additionally, students who had poor health had a greater risk of developing depression, worries, and social withdrawal and lower life satisfaction. Surprisingly, those who had poorer academic achievements were less likely to suffer from depression and worries and had greater life satisfaction. This paradoxical finding may be linked to the pressures and expectations associated with academic success in Korea.

These findings have important implications for addressing unique psychological needs in the context of multicultural adolescents, integrated with parental and socioeconomic factors. More support and policy measures should be taken to increase psychological well-being in this growing demographic segment.

## Limitations

Despite the strengths of this study, several limitations should be acknowledged. While the data were collected through home visits by trained interviewers, they were based on self-reports from the participants and may have response bias. Additionally, the study was based on a cross-sectional survey, so causality could not be confirmed; only associations were identified. Finally, while the results indicate that the country of birth, job, education, and socioeconomic status of parents are associated with the psychological well-being of multicultural adolescents, these associations are not statistically significant.

## Conclusion

This study provides valuable insights into the association of parents’ country of birth and multicultural adolescents psychological well-being in Korea. Key findings revealed that adolescents whose mothers were foreign-born, especially those from China and the Philippines, were more likely to experience higher levels of depression, worries, and social withdrawal and lower life satisfaction. Consequential effects on the psychological well-being of adolescents include the father’s occupation and the use of languages other than Korean at home. These findings suggest that interventions may be beneficial for assisting multicultural adolescents and addressing language barriers, socioeconomic hurdles, and cultural integration. With an in-depth understanding of these specific factors, it becomes easier to formulate practical approaches that positively impact the psychological well-being of multicultural adolescents.

## Supporting information

S1 Data This file contains the full dataset used in the study, including anonymized responses from participants. Variables are coded according to the study methodology.
(XLSX)

S1 Text Readme provides an overview of the supporting information files in this study. **It explains the contents of the dataset and the structure of different sheets within the associated Excel file,** S1 Data.
(PDF)
