# Supplementary material for: Association between parents’ country of birth and multicultural adolescents’ psychological well-being in South Korea: A study on depression, worries, life satisfaction, and social withdrawal
Source: PLOS Ment Health. 2025 Jun 18;2(6):e0000356. doi: 10.1371/journal.pmen.0000356 (PMC12798585; doi:10.1371/journal.pmen.0000356)
Supplement: S1 Text — It explains the contents of the dataset and the structure of different sheets within the associated Excel file, S1 Data. (PDF) [file pmen.0000356.s002.pdf]

Readme: Association between parents' country of birth and multicultural adolescents' psychological well-being in South Korea: A study on depression, worries, life satisfaction, and social withdrawal

1. **Overview**

This dataset contains research data for the study "Association between parents' country of birth and multicultural adolescents' psychological well-being in South Korea: A study on depression, worries, life satisfaction, and social withdrawal", obtained from the National Youth Policy Institute (NYPI). The data is classified as open and is accessible through https://www.nypi.re.kr/archive.

2. **File information**

- File format: XLSX
- Number of sheets: Six data sheets plus results tables

3. **Description of sheets**

- Final codebook: Provides coding schemes used in the study.
- Raw data: Contains unprocessed data collected from NYPI.
- Final data: The cleaned and processed dataset used for analysis.
- Final data (Missed): An additional dataset with adjusted values.
- Variables used: Lists all variables, including types and ranges.
- Average data: Aggregated variables created by averaging similar measures.
- Results tables: Statistical outcomes derived from the study's analysis.

4. **Data usage and methodology**

- The dataset including coding details, variable descriptions, and processed statistical results.
- Users should refer to the final codebook sheet for coding explanation.
- The raw data and final data sheets ensure transparency and reproducibility.

5. **Access and citation**

- This dataset is openly available through the NYPI (https://www.nypi.re.kr/archive).

- If used, please credit the authors and reference the study appropriately. Citation details will be provided once the artilce is accepted.
