# Supplementary material for: Association between parents’ country of birth and multicultural adolescents’ psychological well-being in South Korea: A study on depression, worries, life satisfaction, and social withdrawal
Source: PLOS Ment Health. 2025 Jun 18;2(6):e0000356. doi: 10.1371/journal.pmen.0000356 (PMC12798585; doi:10.1371/journal.pmen.0000356)
Supplement: Attachment — Submitted filename: Response to Reviewers.pdf [file pmen.0000356.s004.pdf]

Rebuttal Letter

Manuscript ID: PMEN-D-25-00105

Journal: PLOS Mental Health

Title: Association between parents' country of birth and multicultural adolescents' psychological well-being in South Korea: A study on depression, worries, life satisfaction, and social withdrawal

Submitted to: Dr. Hanif Rahman

Academic Editor, PLOS Mental Health

Submitted by: Abdullatif Ghafary

Yonsei University

Latif.ghafary@yonsei.ac.kr

2025/05/12

Dear Dr. Rahman,

We are sincerely grateful to the efforts and time taken by the editorial team and reviewers in reviewing our paper, "Association between parents' country of birth and multicultural adolescents' psychological wellbeing in South Korea: A study on depression, worries, life satisfaction, and social withdrawal." The feedback provided have been highly useful to improve our study better and more comprehensively, and more rigorously in its methodology as a whole.

We have carefully reviewed and replied to all comments received during the peer review process. Below, we provide detailed justifications of each point, along with the changes made. We hope these changes are acceptable to the journal and make our manuscript more robust.

Response to Editor:

1. **Data Availability Statement**

   The data for this study were obtain from the National Youth Policy Institute (NYPI) and are open data. It can be accessed through the NYPI archive at https://www.nypi.re.kr/archive. I addition, the dataset is included as supporting information with this manuscript for direct access. A readme file is provided to describe the dataset structure, variables, and coding information.

Response to Reviewer #1:

We appreciate your constructive feedback and insightful comments, which have prompted us to improve the quality of our manuscript. Following are our responses to each one of your concerns:

1. **Formatting and Consistency Issues**
   We have revised the manuscript carefully for consistency in paragraph formatting and for capitalization correction in the title.

2. **Positioning of the Study Focus**
   We have moved the study focus to the end part of the introduction right before the research question and objectives for clarity and logical flow. This revision puts a more definitive emphasis on the study objective such that is explicitly clear to the reader.

3. **Figure Formatting Issues**
   Figures 1 and 2 have been rearranged for proper viewing in the manuscript. In addition, the figure files have been uploaded to the Preflight Analysis and Conversion Engine (PACE) to ensure they are PLOS compliant.

4. **Potential Contradiction in Findings (Lines 172 vs. 228-230)**
   Thank you for pointing out this potential inconsistency to us. We have reviewed our manuscript carefully to provide consistent reporting of life satisfaction by mother's nationalities. The previous statement "Adolescents with foreign mothers generally reported higher life satisfaction" (Line 172) has been changed to reflect the true findings in Table 3, where levels of life satisfaction were comparable by mother's nationalities, with Korean mothers' adolescents reporting slightly higher life satisfaction (78.9%) than Filipino (74.3%) and Chinese (77.1%) mothers' adolescents.
   Moreover, the statement in lines 228-230 regarding depression, worries, social withdrawal, and life satisfaction between adolescent with Filipino and Chinese mothers has been refined to express that, although they showed greater psychological distress, they were not statistically lower in life satisfaction.
   Also, with the small sample size of adolescents with Korean mothers (N=38), we have now stated in our discussion section clearly that this trend should be approached with caution.
   These revisions ensure that the manuscript provides an unambiguous and data-driven perspective of the findings. We appreciate the reviewer's keen eye for detail since this has helped us tighten up the consistency of our conclusions.

Response to Reviewer #2:

we appreciate your candid feedback, which has pushed us to critically evaluate and refine our manuscript. Below are our responses to each of your concerns:

1. **Research Purpose and Literature Review**

   Reviewer's Concern: The research is simply a fishing expedition, a clear research question was not articulated, and there is no literature review to substantiate a clear purpose.

   Response: We respectfully disagree with the characterization of our research as fishing expedition. The manuscript is clearly looking at how parental factors specifically, parent's country of birth is associated with the psychological well-being of multicultural adolescents in South Korea, specifically examining depression, worries, life satisfaction, and social withdrawal. However, we appreciate ensuring that our objectives are well-spelled out in our research. In order to address this, we have enhanced our introduction to particularly position our research question and highlight its importance.

   Regarding the literature review, our study strongly grounded in existing research on parental characteristics and mental health outcomes for adolescents. However, to further strengthen this section, we have expanded our review of existing relevant studies, integrating theoretical and empirical considerations that substantiate the significance of our study.

2. **Causal Inference in a Non-Experimental Design**

   Reviewer's Concern: The analysis reflects patterns that the authors infer to be causal despite a purely non-experimental research design.

   Response: We fully acknowledge the importance of articulating our results correctly in a non-experimental design. Our research was designed to investigate association between parental factors and multicultural adolescents' psychological well-being, never at any point suggesting any causal relationships. For further clarifying, to be accurate and precise in articulating our conclusion in this manuscript, we have thoroughly reviewed the manuscript and adjusted some of the statements where necessary to emphasize that all conclusions are articulated strictly as association rather than causal inference. Specifically, we have reframed language in discussion and results sections to emphasize trends noted rather than implying direct cause-and-effect relationships. We have also strengthened discussion of limitations such that we now are explicitly noting that causal implications cannot be taken from our design. These modifications respond to issues of misinterpretation and delineate the depth of our analytic strategy.

3. **Methodology Transparency**

   Reviewer's Concern: A clear methodology was not articulated. ("A clear research question was not articulated, let alone methodology")

Response: We appreciate the concern, methodological transparency and have endeavored to refine this section for clarity. Because our study builds on secondary data from the Multicultural Adolescent Panel Study (MAPS) conducted by the National Youth Policy Institute (NYPI), independent sampling was not performed. Instead, we extracted and structured the dataset for the purpose of our study. In the manuscript we have indicated clearly how the dataset was used, with a clear description of the manner in which we performed analysis. These revisions provide more transparency and introduce greater rigor to our methodology.

4. **Suitability for Publication**

   Reviewer's Concern: The quality of writing was fine, I just don't think this article belongs in a journal, even with major revisions.

   Response: We respectfully disagree with the assertion that our work is not suitable for publication. Our research addresses an important and growing area of inquiry–multicultural adolescents in South Korea, a population whose psychological well-being deserve scholarly attention. The study is grounded in established literature, employs rigorous analytic approach, and provides thoughtful examination of the intersection between parental background and adolescents psychological well-being. As cultural diversity in South Korea is increasing, our findings offer valuable empirical evidence that is relevant to both academic and policy action.

We sincerely appreciate the time and effort the reviewers and the editorial team have dedicated to evaluating our manuscript. Their valuable feedback has guided us to enhancing clarity and methodological transparency. We have worked diligently to revise the manuscript, addressing study design, literature reviews, and analytical rigor. We hope these revisions sufficiently addresse the concerns raised, strengthen the study's contribution, and reinforce its relevance. We believe the improvements demonstrate the study's value and its suitability for publication.

We look forward to your further assessment and hope our revised manuscript meets the journal's standards for publication.

Sincerely,

Abdullatif Ghafary
